# Arabic Translation of the Weight Self-Stigma Questionnaire: Instrument Validation Study of Factor Structure and Reliability

**DOI:** 10.2196/24169

**Published:** 2020-11-13

**Authors:** Nasser F BinDhim, Nora A Althumiri, Mada H Basyouni, Omar T Sims, Noara Alhusseini, Saleh A Alqahtani

**Affiliations:** 1 Sharik Association for Health Research Riyadh Saudi Arabia; 2 Alfaisal University, College of Medicine Riyadh Saudi Arabia; 3 School of Public Health, Integrative Center for Healthy Aging University of Alabama at Birmingham Birmingham, AL United States; 4 Division of Gastroenterology and Hepatology, Johns Hopkins University Baltimore, MD United States; 5 Liver Transplant Centre, King Faisal Specialist Hospital and Research Centre Riyadh Saudi Arabia

**Keywords:** overweight, stigma, weight self-stigma, Weight Self-Stigma Questionnaire, obesity, Saudi Arabia, questionnaire, validation, reliability, validity

## Abstract

**Background:**

While it is most often associated with its effects on physical health, obesity is also associated with serious self-stigmatization. The lack of a suitable, validated tool to measure weight-related self-stigma in Arabic countries may be partly responsible for the scarcity of literature about this problem.

**Objective:**

This study investigated the reliability and validity of an Arabic version of the Weight Self-Stigma Questionnaire (WSSQ).

**Methods:**

Data on the Arabic-translated version of the 12-item WSSQ were collected using two cross-sectional electronic questionnaires distributed among Saudi nationals through the Sharik Association for Health Research’s database in June 2020. Internal consistency, test-retest reliability, and exploratory factor analysis of the Arabic WSSQ were assessed and compared with the original English version and other translations.

**Results:**

For reliability analysis, 43 participants completed the Arabic WSSQ during two time periods. Internal consistency was α=.898 for the overall survey, α=.819 for the fear of enacted stigma subscale (factor 1), and α=.847 for the self-devaluation subscale (factor 2). The test-retest reliability of the intraclass correlation coefﬁcient was α=.982. In the factor structure analysis, 295 participants completed the questionnaire. The Arabic WSSQ loading of the items was consistent with the original WSSQ, except for the loading of item 9, which was stronger in factor 2 than in factor 1. The two factors accounted for the observed variances of 47.7% and 10.6%.

**Conclusions:**

The Arabic version of the WSSQ has good internal consistency and reliability, and the factorial structure is similar to that of the original WSSQ. The Arabic WSSQ is adaptable for clinicians seeking to assess weight-related self-stigma in Arabic-speaking people.

## Introduction

Obesity is a global health problem with serious complications, including cardiovascular disease, diabetes, and hypertension. It is significantly correlated with cancer, stroke, asthma, and reduced fertility [1]. From 1992 to 2005, the prevalence of obesity in Saudi men aged 25 to 34 years increased from 10.1% to 27.1%, while the prevalence in Saudi women within the same age range increased from 16.1% to 39.5% [2]. Approximately 25% of the Saudi Arabian population suffers from obesity, and around 35% of the total Saudi population is clinically overweight. Along with its impact on physical health, obesity is also associated with psychosocial complications, including stigmatization in health care, employment, education, and other settings.

Stigma enables various forms of discrimination that ultimately deny the individual or group full social acceptance, reduce the individual’s opportunities [3], and fuel social inequalities [4], and it influences population health outcomes by worsening, undermining, or impeding a number of processes, including social relationships, stress, and psychological and behavioral responses [5,6]. Stigma and its effects are classified into two main types: public stigma and self-stigma. Public stigma has been described in terms of stereotypes, prejudice, and discrimination; self-stigma is the awareness of and agreement with public stigma stereotypes and attitudes and the application of those stereotypes to oneself, which undermine self-esteem and self-efficacy [7]. Thus, the availability of recent, high-quality data on health-related stigma or self-stigma is critical for improving interventions and programs to address them, yet such routine data are often lacking, sometimes due to a lack of validated tools for assessment [8].

To date, Arabic-speaking countries have no validated measure of weight self-stigma, constraining researchers’ attempts to determine the prevalence of weight self-stigma and its contribution to adverse health and psychological effects. A reliable, validated Arabic tool would allow researchers to measure the impact of weight-stigma interventions on other health outcomes [9]. Although the Weight Self-Stigma Questionnaire (WSSQ) has wide global application, the translated version must be validated to make sure that it measures what it is intended to and produces results comparable to those of the original version. Translated versions of questionnaires are needed for researchers who intend to collect data among respondents who speak other languages, mainly to compare responses across populations of different languages and/or cultures [10]. In addition, researchers need to make sure that the translated questionnaires are assessing the equivalent construct with an equivalent metric [10].

Widespread clinical uptake of such a questionnaire can inform psychotherapeutic treatment options for overweight and obese patients experiencing psychological stressors from weight self-stigma, which impacts overall patient well-being. Obese patients who receive appropriate psychological treatment tend to lose weight and exhibit a reduction in obesity-related comorbidities [11]. Clinically, the WSSQ is used to diagnose and treat psychological issues associated with self-devaluation and fear of enacted stigma in obese and overweight patients, and such treatment can result in a range of positive outcomes, including decreased emotional overeating and increased health-promoting behaviors [12].

The WSSQ is a self-reported measure of weight-related self-stigma in overweight and obese persons. It has been translated and validated in German, French, Chinese, and Turkish, among other languages [13-16]. To date, a validated Arabic psychometric scale to evaluate weight-related self-stigma has not been adopted by clinicians. This paper reports the development and validation of an Arabic-translated version of the WSSQ. We also compared the internal consistency and test-retest reliability of the Arabic WSSQ with the original English WSSQ and other translations.

## Methods

Analyses of test-retest reliability and exploratory factors were performed for the Arabic-translated version of the WSSQ using data collected via two cross-sectional electronic questionnaires distributed to Saudi nationals through the Sharik Association for Health Research’s database in June 2020.

### WSSQ

The WSSQ is a 12-item Likert-type measure of weight-related self-stigmatization (Table 1). It has two subscales that individually measure (1) weight-related self-devaluation and (2) fear of enacted stigma. The original version of the questionnaire had good psychometrics and a valid preliminary construct. Cronbach α coefficients were acceptable for the full scale (α=.878) and the two subscales (α=.869 and α=.812) [9]. Analyses of principal components revealed a two-factor structure [9]. WSSQ items are rated on a scale of 1 (completely disagree) to 5 (completely agree). Sum scores are calculated for the full scale and each subscale. Items 1 to 6 constitute the self-devaluation subscale (factor 2), and items 7 to 12 constitute the fear of enacted stigma subscale (factor 1). There are no reverse-scored items.

**Table 1:** Weight Self-Stigma Questionnaire items.

Item number	Item
1	I’ll always go back to being overweight.
2	I caused my weight problems.
3	I feel guilty because of my weight problems.
4	I became overweight because I’m a weak person.
5	I would never have any problems with weight if I were stronger.
6	I don’t have enough self-control to maintain a healthy weight.
7	I feel insecure about others’ opinions of me.
8	People discriminate against me because I’ve had weight problems.
9	It’s difficult for people who haven’t had weight problems to relate to me.
10	Others will think I lack self-control because of my weight problems.
11	People think that I am to blame for my weight problems.
12	Others are ashamed to be around me because of my weight.

### Arabic Translation of the WSSQ

Standard backward and forward translation was performed. One nutritionist and 2 research professionals independently conducted the forward translation, and 2 professional translators independently conducted the backward translation. A focus group composed of 8 participants was asked to answer and discuss the questionnaire, and their comments and understanding of each item’s meaning were discussed in the first focus group. Another round of focus group discussions was conducted with a total of 2 focus groups, each with 8 participants. Based on these discussions, the language of the questionnaire items was edited and clarified further, and the final version produced received universal approval among the group members. The final translated items can be found in Multimedia Appendix 1.

### Study Stages

#### Test-Retest Reliability

In June 2020, a group of randomly selected Arabic-speaking adults from the general Saudi population was invited to complete the questionnaire on an electronic form via the Sharik Association for Health Research’s database. The population of this database project is composed of persons who are interested in participating in research projects. An increasing number of participants are registered, now more than 63,000 distributed across the 13 regions of Saudi Arabia [17]. Eligibility was determined automatically via the data collection system. The eligibility criteria were being age 18 years or older and using Arabic as the primary language. Individuals from the participant database who met the eligibility criteria were notified via SMS text message to complete the survey via unique survey links. Three reminders were sent to each potential participant within 1 week. If the participant did not respond, another participant with similar demographics was invited until the required sample size had been reached. The same participants completed the questionnaire again after 1 week. Participants were asked to complete all answers before submitting the questionnaire.

#### Exploratory Factor Analysis

In this stage, a group of randomly selected Arabic-speaking Saudi adults was invited to complete the questionnaire on an electronic form from the Sharik Association for Health Research’s database [17]. Participants completed all answers before submitting the questionnaire. This phase used similar eligibility criteria and a similar recruitment process as was used in the test-retest stage.

### Sample Size

Based on published literature, the recommended sample size for test-retest reliability is 20 to 40 participants [18,19]. The original study that established test-retest reliability of the WSSQ used 44 participants [9]. The recommended sample size for exploratory factor analysis is between 100 and 250 participants, while some studies recommend more than 300 participants to ensure rigorous psychometric validation [20]. The suggested sample size includes a minimum of 2 and a maximum of 20 participants per item. For this study, the recommended minimum sample size was 240 participants based on 20 participants per each of the 12 items on the WSSQ. Thus, the targeted sample size range was 240 to 300 participants.

### Statistical Analysis

Similar to the original WSSQ validation process, internal consistency was assessed using the Cronbach α coefﬁcient, and the test-retest reliability was assessed with the intraclass correlation coefﬁcient. To assess the suitability for conducting an exploratory factor analysis, analyses of the correlation between the scale items were conducted using the Kaiser-Meyer-Olkin sample adequacy measure (nonsignificant results mean the data are suitable for factor analysis) and Bartlett test (significant results mean the data are suitable for factor analysis) [21-24]. To examine the factorial structure of the scales, an exploratory factor analysis using principal factor extraction was performed. The oblique rotation method was used to obtain clear factorial structures and enable comparison with the original study results; an item loading cutoff of greater than 0.15 was adopted, as in the original study, and factors with eigenvalues greater than 1.0 were retained [9]. There were no missing data, as all questions needed to be completed before submitting the questionnaire electronically. All statistical tests were performed using the IBM SPSS statistical package (version 20).

### Ethical Considerations

The ethics committee of the Sharik Association for Health Research, Riyadh, Saudi Arabia, approved this research project according to national research ethics regulations. Participants gave consent electronically, which was recorded by the data collection system.

## Results

### Participants’ Characteristics

Of the 43 participants in study stage 1 (for test-retest reliability), 48.8% (21/43) were male and the mean age was 34.4 years (range 18-66). Of the 295 participants in study stage 2 (for exploratory factor analysis), 48.5% (143/295) were male and the mean age was 33.6 years (range 18-70).

### Reliability

Internal consistency measures for both the subscales and the overall scale were good. The internal consistency of the full Arabic WSSQ was α =.898, and for the subscales—factor 1 and factor 2—internal consistency was α=.819 and α=.847, respectively. These values are similar to those observed in the original English version (α=.878 overall, and α=.869 and α=.812 for the enacted stigma and self-devaluation subscales, respectively) [9]. In the analysis of test-retest reliability, the intraclass correlation coefﬁcient was α=.982.

### Factor Structure

Correlation coefﬁcients among the 12 items were all 0.3 or higher, except those for item 12, which were below 0.3 for some other items. The Kaiser-Meyer-Oklin value was 0.891, and values for Bartlett’s test of sphericity reached statistical signiﬁcance (*P*<0.001).

**Figure 1:**
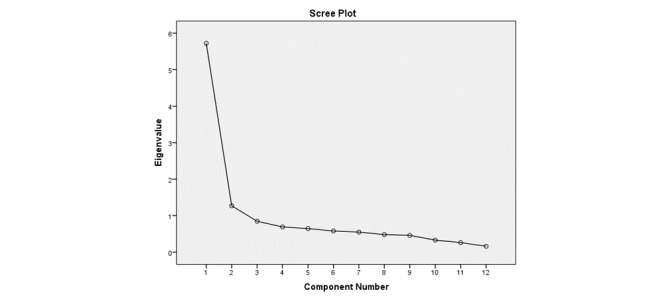
Scree plot for principal component analysis: Comparison of the 12 components of the Arabic WSSQ to evaluate statistical significance of components using a scree test to determine eigenvalues.

An analysis of principal components revealed two components that explained 47.7% and 10.6% of the variance. The scree plot (Figure 1) revealed a break after the second component. Oblique rotation (Table 2) was performed to compare the results with the original scale, and identical variance explanations of 47.7% and 10.6% were found.

Test-retest reliability/stability with the intracorrelation coefﬁcient was high. Exploratory factor analysis revealed that loading of the items for the Arabic WSSQ was consistent with that of the original English WSSQ, except for the loading of item 9 (Table 2), which loaded positively in both factors but more strongly in factor 2 than in factor 1 [9].

**Table 2:** Weight Self-Stigma Questionnaire items: item-scale correlations and factor loadings from principal components analysis with oblique rotation.

	Arabic version			English (original) version		
Item	Item-scale correlation	Factor 1	Factor 2	Item-scale correlation	Factor 1	Factor 2
1	0.65	0.26	0.69	0.55	Not reported	0.55
2	0.73	0.54	0.73	0.47	Not reported	0.66
3	0.75	0.54	0.72	0.71	Not reported	0.69
4	0.81	0.50	0.82	0.71	Not reported	0.81
5	0.53	0.16	0.65	0.63	Not reported	0.69
6	0.68	0.33	0.77	0.68	Not reported	0.79
7	0.71	0.68	0.52	0.68	0.67	Not reported
8	0.66	0.79	0.36	0.66	0.86	Not reported
9	0.62	0.32	0.69	0.67	0.72	Not reported
10	0.62	0.77	0.61	0.78	0.79	Not reported
11	0.77	0.79	0.61	0.66	0.72	Not reported
12	0.40	0.70	0.15	0.67	0.82	Not reported

## Discussion

### Principal Findings

This study involved translating the WSSQ into Arabic and testing the reliability and validity of the Arabic-translated version. The Arabic WSSQ demonstrated good psychometric properties, which were congruent with the original English version of the WSSQ. The internal consistency of the Arabic WSSQ and its subscales (ie, fear of enacted stigma and self-devaluation) was good: α=.898, α=.819, and α=.847, respectively. These values were consistent with the original English version of the WSSQ and its subscales: α=.878, α=.869, and α=.812, respectively [9]. Test-retest reliability of the Arabic WSSQ was high at 0.982. Exploratory factor analysis showed that the loading of the majority of the questionnaire items (except item 9) was consistent with the original English version of the WSSQ.

We searched PubMed for any published research articles from Saudi Arabia about weight stigma in general and found no related articles. The lack of research conducted on weight-related stigma in Saudi Arabia may be the result of a lack of validated tools for assessing this topic. The validation of such a tool, as presented in this study, provides an initial means to fill that gap. More tools to measure weight-related stigma for different age groups and dimensions (eg, public weight stigma) are needed to expand this research field and generate more understanding about the impact of weight self-stigma on quality of life and psychological stress. Increased use of the Arabic WSSQ has the potential to close the gap between the well-known high prevalence of obesity and the scarcity of knowledge on the frequency and severity of weight self-stigma among Saudi adults. To enable the expansion of WSSQ utilization to Arabic-speaking countries, we have provided the first Arabic translation of the WSSQ.

### Study Limitations and Strengths

This study was not without notable limitations and strengths. The utility of the Arabic-WSSQ is not generalizable to children, as the study included only adults. A larger sample size is needed to assess the cross-cultural validity of this scale using conﬁrmatory factorial analysis. Also, convergent validity was not assessed, and future studies are needed to assess correlations between the Arabic WSSQ and other scales that measure weight self-stigma.

However, the study had a high response rate among participants. The study’s sample size was in line with published sample size recommendations for psychometric validation and was consistent with the sample size of the initial study that established the psychometric properties of the original English version of the WSSQ [9]. To our knowledge, this is the first Arabic translation of the WSSQ and the first demonstration of its psychometric properties among Arabic-speaking Saudi adults. The Arabic WSSQ not only allows researchers and clinicians to assess weight self-stigma among Arabic-speaking adults, but it provides researchers and clinicians with an empirical measure to assess the effectiveness of interventions to reduce weight self-stigma.

### Conclusion

The Arabic WSSQ has good internal consistency and reliability. The factorial structure is similar to the original English WSSQ, which endorses its value for use in cross-cultural studies. The Arabic WSSQ appears to be a reliable measure for assessing weight-related self-stigma in Arabic-speaking people.

## Appendix

Multimedia Appendix 1 Arabic translation and validation of the Weight Self-Stigma Questionnaire.

## Abbreviations

WSSQ: Weight Self-Stigma Questionnaire
