# Supplementary material for: Arabic Translation of the Weight Self-Stigma Questionnaire: Instrument Validation Study of Factor Structure and Reliability
Source: JMIR Form Res. 2020 Nov 13;4(11):e24169. doi: 10.2196/24169 (PMC7695527; doi:10.2196/24169)
Supplement: Multimedia Appendix 1 [file formative_v4i11e24169_app1.docx]

Arabic Translation and Validation of the Weight Self-Stigma Questionnaire: Factor Structure and Reliability

| **Item in Original English Scale** | **Translated Arabic Item** |
| --- | --- |
| 1. I’ll always go back to being overweight | دائما سأعود لسمنة بعد خسارة بعض الوزن |
| 2. I caused my weight problems | انا السبب في مشاكل وزني |
| 3. I feel guilty because of my weight problems | أشعر بالذنب بسبب مشاكل وزني |
| 4. I became overweight because I’m a weak person | أصبحت سميناً بسبب ضعف إرادتي |
| 5. I would never have any problems with weight if I were stronger | لن أواجه أي مشاكل مع وزني لو كنت امتلك إرادة قوية |
| 6. I don’t have enough self-control to maintain a healthy weight | ليس لدي الإرادة القوية للمحافظة على وزني المثالي |
| 7. I feel insecure about others’ opinions of me | اشعر ان الاخرين يجاملونني برأيهم تجاه وزني؟ |
| 8. People discriminate against me because I’ve had weight problems | يعاملني الناس بتمييز بسبب مشاكل وزني |
| 9. It’s difficult for people who haven’t had weight problems to relate to me | من الصعب على الأشخاص الذين لا يعانون من مشاكل في الوزن أن يتفهموا شعوري |
| 10. Others will think I lack self-control because of my weight problems | يرى الأخرين أن سبب زيادة وزني هو عدم قدرتي على التحكم بسلوكياتي؟ |
| 11. People think that I am to blame for my weight problems | يلومني الأخرين بأنني السبب في زيادة وزني؟ |
| 12. Others are ashamed to be around me because of my weight | يخجل الاخرون من مرافقتي بسبب وزني ؟ |
